# Volume Threshold for Chest Tube Removal: A Randomized Controlled Trial

**DOI:** 10.5249/jivr.v1i1.5

**Published:** 2009-07

**Authors:** Mohammad Ali Hessami, Farid Najafi, Sajad Hatami

**Affiliations:** ^*a*^Department of Surgery, Kermanshah University of Medical Science, Iran.; ^*b*^School of Population Health/ Kermanshah Health Research Center (KHRC), Kermanshah.

## Abstract

**Background:**

Despite importance of chest tube insertion in chest trauma, there is no general agreement on the level of daily volume drainage from chest tube. This study was conducted to compare the effectiveness and safety of chest tube removal at the levels of 150 ml/day and 200 ml/day.

**Methods:**

Eligible patients (138) who needed replacement of chest tube (because of trauma or malignancy) were randomized into two groups; control (removal of chest tube when drainage reached to 150 ml/day) and trial (removal of chest tube at the level of 200 ml/day). All patients received standard care during hospital admission and a follow-up visit after 7days of discharge from hospital. Patients were then compared in terms of major clinical outcomes using chisquared and t-test.

**Results:**

From the total of 138 patients, 70 and 68 patients were randomized to control (G150) and trial (G200) group, respectively. Baseline characteristics were comparable between the two groups. Although the trial group had a shorter mean for length of hospital stay (LOS) (4.1 compared to 4.8, p=0.04), their differences in drainage time did not reach to the level of statistical significance (p=0.1). Analysis of data showed no statistically significant differences between the rate of radiological reaccumulation, thoracentesis and decrease in pulmonary sounds (auscultatory), one week after discharge from hospital.

**Conclusions:**

Compared to a daily volume drainage of 150 ml, removal of chest tube when there is 200 ml/day is safe and will even result in a shorter hospital stay. This in turn leads to a lower cost.

## Introduction

Injuries are one of the leading causes of burden of disease around the world and it is expected to rise dramatically by the year 2020.^1^ It has been reported that chest trauma is a component of 30% of all cases of trauma. In fact, thoracic procedures are major parts of general surgery.^2,3^ Chest tube (CT) insertion is often indicated for management of pneumothorax haemothorax and/or pleural effusion either due to a benign or malignant condition. Subsequent management of CT is related to different factors such as primary reason for insertion of CT, whether or not the patients is mechanically ventilated and whether patient has a pulmonary resection.^2,3^ However, duration of hospital admission for patients with CT is directly related, at least partly, to daily fluid drainage.^2,5^

Although indications for insertion of CT and the relevant techniques are generally accepted among surgeons, an indication for its removal is often controversial.^5,6^ In fact CT management in general, and CT removal time specifically, are often based on the physicians' experiences and training.The need for an ideal CT management algorithm is highlighted by observed differences in recommendations for volume threshold for timing of chest tube removal.^3-4,7^

While premature CT removal may lead to fluid reaccumulation and a longer hospital stay with higher complications and costs, delay in CT removal may run the risk of relevant complications which in turn brings about the same consequences. With limitations in financial health resources in all developed and developing countries, establishment of a safe and practical volume threshold for removal of CT is an important mission. This randomized clinical trial was conducted to determine the appropriate volume threshold for removing CT. We hypothesized that the safety of chest tube removal with a daily drainage of 200 ml/day is comparable with more generally accepted level of 150 ml/day.

## Methods

From December 2007 to April 2008, all patients with any ages sustaining blunt or penetrating chest trauma as well as those with non-infected pleural effusion due to any malignancies or other benign cause who required CT insertion were evaluated for inclusion in this study. Those who had heart failure, nephritic syndrome, chronic renal failure and cirrhosis were excluded from recruitment. One hundred and forty two patients were recruited from all relevant teaching hospitals (Imam Reza and Talegani Hospital) in Kermanshah, the central city of Kermanshah province in western part of Iran. Of 142 patients, four were excluded due to developing complication as a result of sustained bronchopleural fistula which in turn prevents full lung re-expansion. The patients were assigned to two groups: group 1 (control)-with fluid drainage of 150 ml/day; and group 2-(intervention) with fluid drainage of 200 ml/day using a computer generated randomization list. The process of randomization and its implementation was supervised by the study epidemiologist. Only those who signed written consent form were included in this study. Assuming standard deviation of two days mean length of hospital stay (LOS), our study has a power of 0.8 to detect a significant differences of 1 day (p=0.05, two-sided) in the mean of lOS.

**Study intervention**

For all patients CTs were inserted in standard manner and under aseptic technique followed by surgical dressing. All chest tubes were inserted through 7th intercostal space at axillary line. To evaluate the resolution of the pneumothorax and or pleural effusion, a chest x-ray was obtained one hour after the insertion. Additional chest x-rays were obtained based on evaluation of patients until the CT removal. In addition, all CTs were removed when patients were at maximal inspiratory effort and the wounds were dressed with Vaseline or Xerofrom gauze dressing. A follow-up chest x-ray was obtained 8 to 10 hours after the removal in order to evaluate radiologic reaccumulation rate and chest expansion. Other complications were then investigated one week after patient discharge during the follow-up visit in the surgery clinics.

**Study measures**

For the purpose of this study, the two groups were compared based on the following variables: LOS, occurrence of radiologic reaccumulation, any need for thoracentesis, drainage time, and if there were any decreases in pulmonary sound, one week after discharge from hospital. We regarded a patient as positive for radiologic reaccumulation if there was a report of at least blunting in costophrenic angle in standing chest x-ray (which indicates a reaccumulation of at least 150 ml of fluid) taken 8-10 hour after removal of CT.

Thoracentesis was done for patients who had a positive radiologic reaccumulation as well as a respiratory complain such as shortening of breath.

The period between CT insertion and its removal was defined as drainage time. Decrease in pulmonary sound was confirmed by physician's diagnosis. Length of hospital stay was regarded as the period between CT insertion and patients' discharge.

**Statistical analyses**

Using STATA version 8, we summarized the characteristics of patients using proportions or means and standard deviations where appropriate. Baseline characteristics of patients in two groups were compared, using t-test (for continuous variables) and chi-squared test (for categorical variables). These tests were used to demonstrate if the two groups were similar in respect to demographic and other baseline characteristics before assignment to study groups. To compare the outcome variables between the control and trial, chi-squared test was used. The null hypothesis was rejected for p<0.05.

## Results

**Figure 1:**
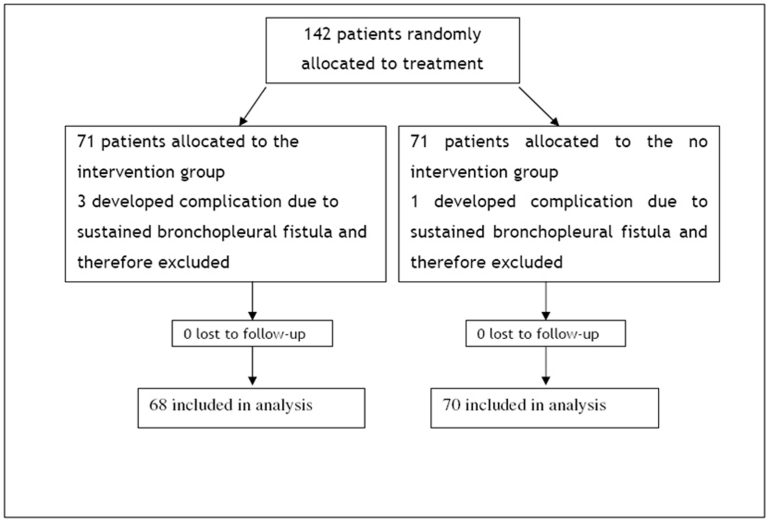
Figure 1:Flow diagram of a trial of the removal of chest tube on the basis of volume drainage

After exclusion of four patients, a total of 138 patients with pleural effusion or pneumothorax met the inclusion criteria (Figure 1). Demographic data is presented in Table 1  for both groups. Two groups are comparable in terms of mean age, sex and cause of pleural effusion (p>0.05). The average age for total sample was 44.0+ 16.8 and only 38 patients (26.8%) were female.

**Table:** Table 1: Characteristics of patients* in control and group

Characteristics	Control (N=70)	Trial (N=68)	p value
Age (mean±SD)	44.1±17.1	44.0±16.7	0.96
Sex (female)	32.9	20.6	0.10
Cause (trauma)	57.1	54.4	0.75

* All values (except for age) are presented in percentage

**Patients' outcomes**

Of 138 patients, 11 patients had appreciable radiologic fluid reaccumulation diagnosed by physical examination and confirmed by radiology, after removal of CT and during hospital stay. All of these patients were those with malignancy. Of these 11 patients, six (55%) needed thoracentesis during their hospital course. After seven days of follow-up, seven patients presented with decreased pulmonary sounds during their physical examination. Majority of these patients (6 patients) were mainly those with malignancy.

Analysis of data from all patients indicated no significant differences between the two groups in the number of patients who were positive for radiologic reaccumulation, or in need of thoracentesis (p<0.05). In addition, seven days after the removal of CT, there was no significant difference between the proportion of patients with decreased pulmonary sound in trial and control group. Patients in control group stayed longer in hospital (4.8 day compared to 4.1 day) (Table 2).

**Table:** Table 2: **Comparison between patients' outcomes**

Characteristics	Control (N=70)	Trial (N=68)	p value
Length of hospital stay (mean±SD)	4.8±1.7	4.1±1.8	0.04
Drainage time (mean±SD)	3.8±1.5	3.4±1.6	0.1
Radiologic reaccumulation (%)	7.1	8.8	0.62
Thoracentesis (%)	4.3	4.3	0.97
Decrease pulmonary sound (%)	5.7	4.4	0.72

## Discussion

The present study has shown that the removal of chest tube when the amount of daily drainage was 200 ml is as safe as when it was 150 ml. This would imply a shorter hospital stay and therefore lower hospital costs and complications.

Although chest tube is a commonplace among patients injured by trauma or those who are in advanced stage of cancers involving lung or pleura, there is little information about the best method for managing CT.^4-10^ Even there are different recommendations from surgical text books, therefore,^2,3^ practitioners use different strategies based on their own experience.

The present study is one of the few investigations attempting to determine the best volume threshold for chest tube removal among those with no infectious pleural effusion. In our sample, the outcomes were similar in terms of proportion of thoracentesis, radiologic reaccumulation and reduction in pulmonary sound. Although inconsistent with the previous report by Younes et al (2002),^4^ we found a shorter hospital stay for patients in trial group, other findings are in line with that report. A recent study has shown that even the removal of CT at drainage up to 450 ml/day is safe.^11^ Such practice will result in the earlier patient ambulation and fewer chest x-rays after insertion of CT and finally shorter hospital stay.

**Limitations and strengths**

Although we managed to prove a difference in the length of hospital stay between the two groups, the available data had limited power to examine the differences in other outcomes. Similar to the previous report,^4^ our study examined short term complications after the removal of chest tube. In fact, some surgeons believe that the insertion of CT for a longer period might lead to a lower incidence of retained haemothorax or pneumothorax. Our study does not support such assertion. In the current study, we did not measure the severity of condition (trauma and/or malignancy) causing a hospital admission and insertion of CT. We believe that it is less likely that such variable rather than daily drainage could determine the appropriate time for CT removal. In fact, our findings showed that when drainage reach 200 ml/day, it is safe to remove the chest tube irrespective of the course of pleural effusion or pneumothorax.

Equal distribution of demographic factors, causes of pleural effusion, assessment of outcomes by only two surgeons and inclusion of both pleural effusion due to malignancy and trauma, are all factors that increase the validity of our findings.

Future studies should recruit a larger sample size with longer follow-up and higher presentation of females. Acceptance of a higher threshold for daily volume drainage will require further investigations.

## Conclusions

In keeping with previous report,^4^ this study demonstrated that using a daily drainage threshold of 200 ml for chest tube safely decrease length of stay at hospital. Therefore the use of lower threshold contradicts the idea of further complications because of early CT removal. In most developed and developing countries facing large burden of trauma and road traffic injury,^12^ with incurring increased health care cost,^13^ current practices recommend to accommodate the available evidence regarding the management of chest tube removal.
